# A review on experimental surgical models and anesthetic protocols of heart failure in rats

**DOI:** 10.3389/fvets.2023.1103229

**Published:** 2023-03-27

**Authors:** Ahmed Farag, Ahmed S. Mandour, Hanan Hendawy, Asmaa Elhaieg, Ahmed Elfadadny, Ryou Tanaka

**Affiliations:** ^1^Department of Veterinary Surgery, Faculty of Veterinary Medicine, Tokyo University of Agriculture and Technology, Fuchu, Japan; ^2^Department of Surgery, Anesthesiology, and Radiology, Faculty of Veterinary Medicine, Zagazig University, Zagazig, Egypt; ^3^Department of Animal Medicine (Internal Medicine), Faculty of Veterinary Medicine, Suez Canal University, Ismailia, Egypt; ^4^Department of Animal Internal Medicine, Faculty of Veterinary Medicine, Damanhur University, Damanhur El-Beheira, Egypt

**Keywords:** heart failure, rats, surgical models, anesthesia, myocardial infarction

## Abstract

Heart failure (HF) is a serious health and economic burden worldwide, and its prevalence is continuously increasing. Current medications effectively moderate the progression of symptoms, and there is a need for novel preventative and reparative treatments. The development of novel HF treatments requires the testing of potential therapeutic procedures in appropriate animal models of HF. During the past decades, murine models have been extensively used in fundamental and translational research studies to better understand the pathophysiological mechanisms of HF and develop more effective methods to prevent and control congestive HF. Proper surgical approaches and anesthetic protocols are the first steps in creating these models, and each successful approach requires a proper anesthetic protocol that maintains good recovery and high survival rates after surgery. However, each protocol may have shortcomings that limit the study's outcomes. In addition, the ethical regulations of animal welfare in certain countries prohibit the use of specific anesthetic agents, which are widely used to establish animal models. This review summarizes the most common and recent surgical models of HF and the anesthetic protocols used in rat models. We will highlight the surgical approach of each model, the use of anesthesia, and the limitations of the model in the study of the pathophysiology and therapeutic basis of common cardiovascular diseases.

## 1. Introduction

Heart failure (HF) is a leading cause of death worldwide. The mortality rate of HF is very high, with ~50% of patients dying within 5 years of their initial diagnosis, which is higher than the fatality rate of most cancers. The most recent World Health Organization estimates that cardiovascular disorders kill 17.9 million people each year, accounting for ~31% of all global deaths (1), and there is a significant economic burden due to the rising prevalence of HF in industrialized countries. The enhancement in treatment for acute myocardial infarction (MI), which has reduced the mortality rate but not morbidity and is based on the rate of survivors, is at least partly responsible for this increase. Additional factors include an increased prevalence of comorbidities, which accelerate the progression of HF. Therefore, it is essential to modify these risk factors and develop new treatment strategies for HF patients (2).

The study of HF requires dependable animal models to evaluate severe changes and pharmacodynamic interactions in the structure and function of the injured myocardium and to pursue its progression to HF. In recent decades, researchers have used small animal models to better understand the pathophysiology of HF and develop more effective strategies for managing patients with congestive HF (3). Therefore, this review aims to describe the different surgical rat models commonly used for the induction of HF and to identify the most reliable anesthetic regimes required for these procedures.

### 1.1. Circulatory system in rats

**Figure 1:**
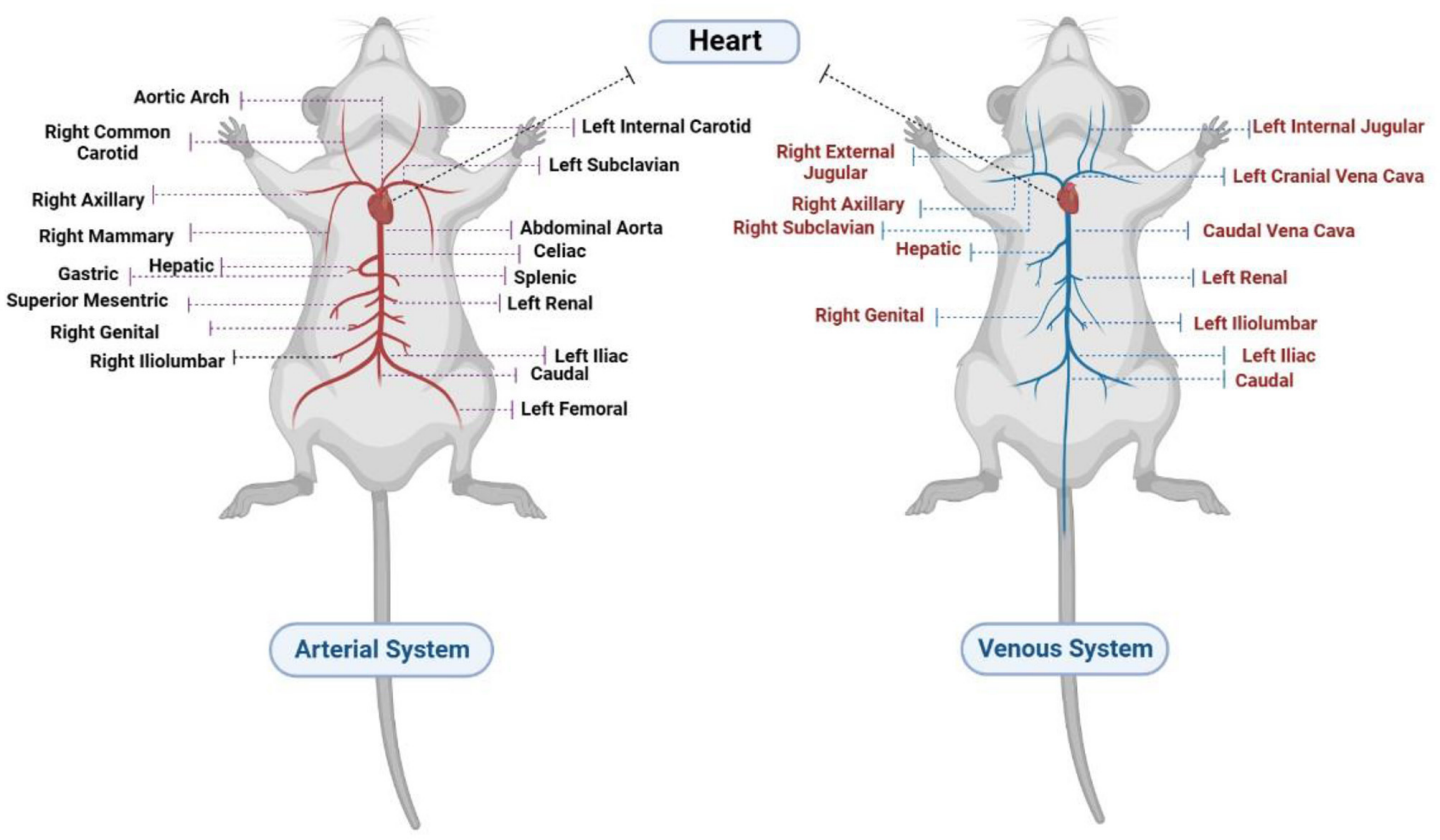
Circulatory system in rats.

Rats are mammals belonging to the Muroidea rodent superfamily. The cardiac, pulmonary, and systemic circulatory systems, as well as their valves, are similar to those of humans. The rat heart has four chambers. On the right side of the aortic arch, the brachiocephalic trunk branches into the right common carotid artery and the right subclavian artery. The left common carotid artery is located in the anterior part of the aortic arch, while the left subclavian artery is located to its left (4, 5). Furthermore, the internal mammary arteries supply coronary blood to the right and left atria (5). The rat also has no true equivalent of a circumflex artery besides a small artery such as a ramus intermediate (6); the arterial and venous systems in rats are illustrated in detail in Figure 1.

### 1.2. Anesthesia in rats

Anesthesia is an essential aspect of laboratory animal research to minimize pain and stress during experimental procedures and is also essential to ensure the reproducibility of experimental results. Appropriate anesthesia administration is crucial in achieving success in surgical experiments (7, 8). Here is a general overview of anesthesia in rats, and the various detailed protocols are explained in Table 1.

**Table 1:** Different anesthetic protocols used for the induction of experimental surgical models of heart failure in rats.

**Anesthetic protocols**	**Surgical models**	**References**
A mixture of ketamine hydrochloride and xylazine hydrochloride (intraperitoneal injection)	Myocardial infarction (MI) model (80 mg/kg ketamine and 10 mg/kg xylazine)	(9, 10)
	Cryoinjury-induced MI model (100 mg/kg ketamine and 10 mg/kg xylazine)	(11)
	Ischemia-reperfusion (IR) model (40 mg/kg ketamine and 10 mg/kg xylazine)	(12)
	Arteriovenous shunt (AVS) model (90 mg/kg ketamine and 10 mg/kg xylazine)	(13, 14)
	Aortic regurgitation (AR) model (50 mg/kg ketamine and 10 mg/kg xylazine)	(15, 16)
Sodium pentobarbital (intraperitoneal injection)	MI model (50 mg/ kg sodium pentobarbital)	(17–19)
	Cryoinjury-induced MI model (50 mg/ kg sodium pentobarbital)	(20)
	IR model (50–60 mg/kg sodium pentobarbital)	(21–23)
	Aortic constriction (AC) model (40 mg/kg sodium pentobarbital)	(24, 25)
	AR model (50 mg/ kg sodium pentobarbital)	(26, 27)
	PAB model (50–60 mg/ kg sodium pentobarbital)	(28–31)
	AVS model (50 mg/ kg sodium pentobarbital)	(32, 33)
	Two kidneys, one clip (2K1C) model (40 mg/kg sodium pentobarbital)	(34–36)
Chloral hydrate 10 % (intraperitoneal injection)	MI model	(37, 38)
	IR model (350 mg/kg chloral hydrate 10 %)	(39, 40)
	AC model (300 mg/kg chloral hydrate 10 %)	(41, 42)
	2K1C model (0.3 ml/100 g 10% chloral hydrate)	(43)
Isoflurane (inhalational anesthesia)	MI model (induction: 5%, maintenance: 2.5%)	(44)
	AC model (induction: 4%, maintenance: 2.5%)	(45)
	AVS model (induction: 4%, maintenance: 1.5%)	(32, 46)
	AR model (maintenance: 1.5%)	(47)
	PAB model (induction: 4% isoflurane in a mixture of 50% O_2_ and 50% N_2_O)	(48)
A mixture of ketamine, xylazine, and acepromazine.	MI model (50 mg/kg ketamine, 4 mg/kg xylazine, and 1 mg/kg acepromazine).	(49)
Intraperitoneal injection of sodium pentobarbital followed by an Intramuscular administration of ketamine hydrochloride.	Cryoinjury-induced MI model (30 mg/kg sodium pentobarbital and 22 mg/kg ketamine hydrochloride)	(50)
Intramuscular ketamine injection followed by an intraperitoneal injection of pentobarbital.	Cryoinjury-induced MI model (22 mg/kg ketamine and 30 mg/kg pentobarbital).	(51)
Diethyl ether (inhalational anesthesia)	Cryoinjury-induced MI model	(52)
A mixture of ketamine and medetomidine intramuscular injection	2K1C model (60 mg/k ketamine and 250 μg/kg medetomidine)	(53)
Ketamine intraperitoneal injection	2K1C model (90 mg/kg ketamine)	(54, 55)
A mixture of injectable and inhalational anesthesia ketamine hydrochloride and isoflurane	AVS model (10 mg per rat ketamine hydrochloride and subsequently anesthetized using 5% isoflurane for the first minute followed by 2–3% during the remainder of the surgery).	(56)
A mixture of ketamine and midazolam	AVS model	(57)
Methohexital sodium intraperitoneal injection	AVS model (50 mg/kg methohexital sodium).	(58)
A mixture of pentobarbital and xylazine intraperitoneal injection	PAB model (50 mg/kg pentobarbital and 5 mg/kg xylazine)	(59)
A mixture of medetomidine- midazolam-butorphanol (MMB) and isoflurane followed by atipamezole	MI model MMB (0.3/5.0/5.0 mg/kg/SC) with isoflurane 1% encountered by atipamezole 1.0 mg/kg/SC	(60)

#### 1.2.1. Inhalant anesthesia

Isoflurane and sevoflurane are commonly used inhalant anesthetics in rats. They are administered through a mask or nose cone to induce and maintain anesthesia. The dose and concentration of anesthetic gas can vary based on the species, weight, and age of the rat, as well as the procedure being performed (61, 62).

#### 1.2.2. Intraperitoneal injection

A combination of ketamine (50–100 mg/kg) and xylazine (5–10 mg/kg) is a commonly used anesthetic protocol for intraperitoneal injections in rats. This method provides a rapid onset of anesthesia and is often used in short procedures (63, 64).

#### 1.2.3. Intramuscular injection

A combination of ketamine (50–100 mg/kg) and xylazine (5–10 mg/kg) is a commonly used anesthetic protocol for intramuscular injections in rats. This method also provides a rapid onset of anesthesia and is often used as a backup when inhalant anesthesia is impossible (65).

#### 1.2.4. Intravenous injection

Propofol (2–4 mg/kg) is a commonly used anesthetic for intravenous injections in rats. This method provides rapid and controlled induction of anesthesia and is often used for more invasive procedures (66).

Unfortunately, some countries have prohibited some anesthetic drugs and classified them as narcotics; for instance, Ketamine is currently classified as a narcotic medication in Japan, and numerous other countries have reinforced limitations on its purchase, possession, and related record-keeping methods (67). These decisions represent a big obstacle to their researchers and thus increase the challenges of finding alternative anesthetic protocols. Therefore, we have recently published a paper describing a novel protocol for induction of general anesthesia in rats for cardiac surgery using a mixture of injectable and inhalation anesthesia along with antagonists (60).

It is important to note that the correct protocol can vary greatly depending on the individual animal and the procedure being performed. Additionally, close monitoring of an animal's vital signs, such as heart rate, respiratory rate, and body temperature, is crucial during anesthetic procedures to ensure the safety and wellbeing of the animal.

## 2. Surgical models

### 2.1. Myocardial infarction

Coronary circulation is the main supply of blood to the cardiac tissues, and effective coronary circulation is crucial for the health of the myocardium. Constriction or blockage of one or more branches of the coronary artery is life-threatening and may cause irreversible heart damage and MI; therefore, MI is the main type of ischemic heart disease, characterized by unbalanced ischemia and myocardial necrosis (68, 69).

Despite significant improvements in prognosis, acute myocardial infarction remains the most severe manifestation of coronary artery disease, affecting over seven million people worldwide and contributing to over four million fatalities annually in Northern Asia and Europe (70, 71). MI is described as necrosis of the cardiac muscle cells caused by a prolonged lack of oxygen supply. Because of the decrease in blood circulation, there is insufficient oxygen and nutrition supply to fulfill tissue demand. As a result, cardiomyocyte death occurs (72). Furthermore, in chronic situations, MI may worsen hemodynamics, resulting in patient death. When an acute MI occurs, the patient typically has extensive pain in the chest, upper abdomen, and other regions for at least 20 mins, accompanied by symptoms such as dyspnea (73). Following MI, myocardial cells undergo acute necrosis, and fibrotic scars form during the repair phase. The formation and build-up of fibrotic scars over time may damage the structure and function of the heart (74).

### 2.2. Surgical methods of MI

#### 2.2.1. Coronary artery ligation

CAL in a rat model is a research technique commonly used to induce MI (heart attack) in rats to study the pathophysiology of the disease, test potential therapeutic interventions (75, 76), evaluate the efficacy of stem cell therapy (9), investigate changes in BM-MSCs *in vivo* and their ability to differentiate into contractile myocytes (77), and explore the effect of autophagy on acute MI and its mechanism in rats (78).

Permanent CAL results in total blood flow blockage and irreversible hypoxia, leaving most of the area at risk of infarction and a massive and permanent scar in the myocardium. This damaged area is susceptible to pathological remodeling, which leads to the progression of HF. Furthermore, the site of the artery blockage influences the size of myocardial ischemia, with ligation closer to the heart's base causing more severe damage. The use of a well-proven procedure performed by a qualified surgeon lowered the variation in infarct size based on the ligation site (79).

##### 2.2.1.1. Surgical technique

The procedure began with the injection of an anesthetic drug into the animal, followed by the use of a mechanical ventilator to secure the airway. A left thoracotomy was performed, the heart was rapidly exposed, and the initial ligation site was determined (80).

**Figure 2:**
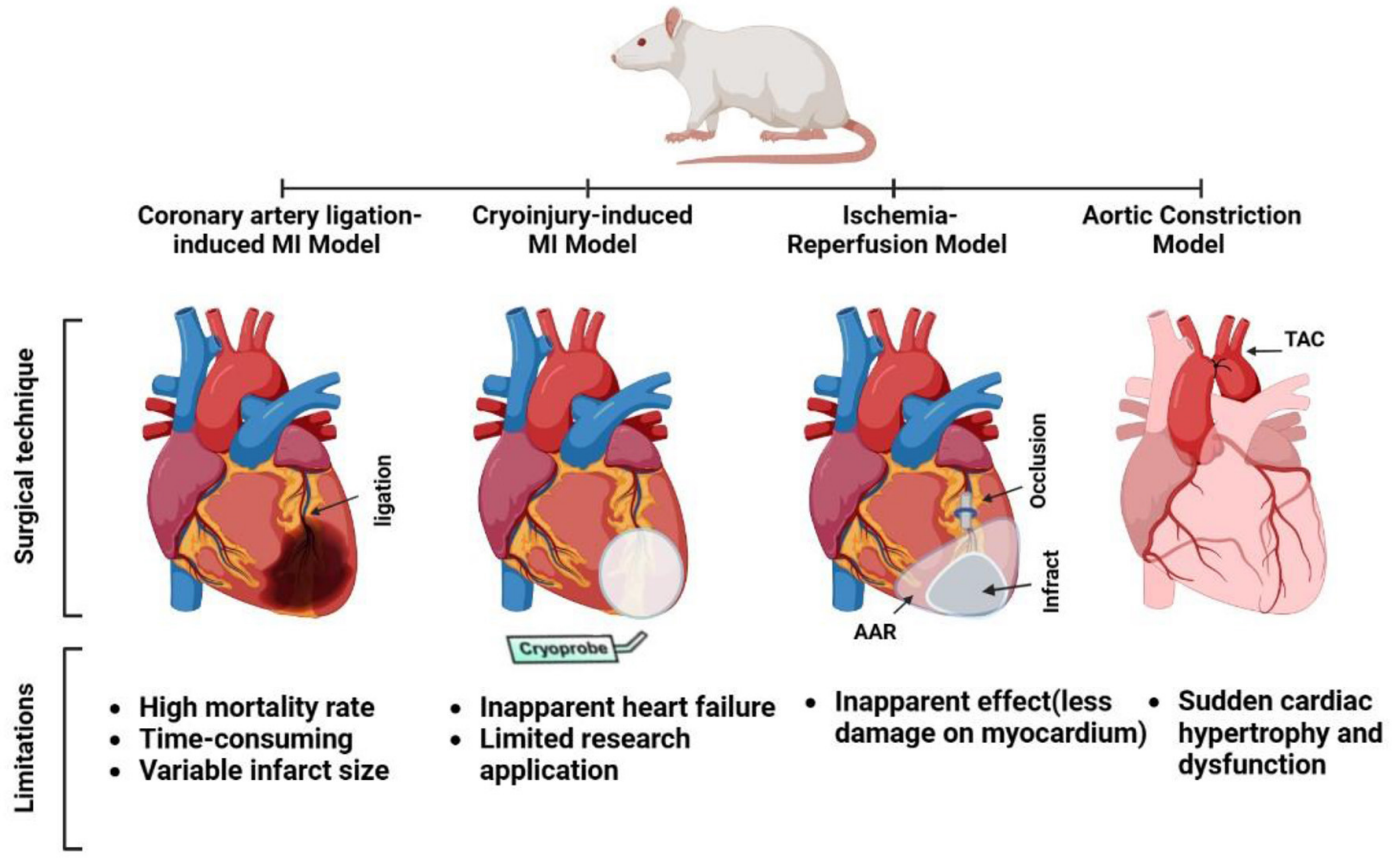
Surgical techniques and limitations for some experimental rat models of heart failure (coronary artery ligation myocardial infarction [MI], cryoinjury MI, ischemia-reperfusion, and aortic constriction models).

Once the site of ligation of the left anterior descending coronary artery (LAD) was identified, a cotton earbud was gently pressed onto the artery slightly below the site of ligation, immobilizing the heart, while simultaneously making the artery noticeable and easy to recognize. A non-absorbable ligature passes below the LAD and is secured with three knots using a tapered atraumatic needle. Blanching and cyanosis of the anterior wall of the left ventricle, as well as enlargement of the left atrium, are signs of successful ligation. Due to direct vision and observation of the process and targeted area of infarction, CAL provides accurate time, location, and extent of the coronary event. The ribs and muscles were closed with dissolvable sutures, with a small gap left to aspirate any remaining air in the thorax, and air was aspirated to keep the lungs from collapsing. At the time of closure, the muscle and skin stitch sites were treated with neomycin powder and betadine, respectively. Before extubation, the lungs were deflated by submerging the exit tube connected to the endotracheal tube in an underwater seal with adequate postoperative care (Figure 2) (9).

Extubation was conducted before the rats were fully awake, and a 1 mL syringe was used to carefully suction the endotracheal catheter to prevent bronchial occlusion due to heavy mucus. The rats were then placed in a recovery cage with an oxygen source for around 30 min. Analgesia (0.025 mg/kg body weight subcutaneously every 12 h) was planned for up to 72 h (81).

##### 2.2.1.2. Limitations

These procedures are reported to have a mortality rate of more than 50% due to malignant ventricular tachycardia in the acute phase. Furthermore, infarctions are usually mild (averaging 21% of the left ventricle), which may be due to the large amount of sub-pericardial collateral circulation in this species. Consequently, only minor hemodynamic changes were observed (82). Furthermore, creating the model is time-consuming and has been increasingly criticized in terms of animal protection (83). Reichert et al. (84) mentioned that the main limitation of this technique is the risk of postoperative mortality, mostly caused by the occurrence of cardiac arrhythmias, hemorrhage, and pneumothorax.

However, the MI model using the ligation technique produces a wide variation in infarct size (85, 86). According to Pfeffer et al. (87), the extent of the infarct ranges from 8 to 65%. Widely varying results have minimal statistical significance and limited utility, and infarct size is a significant predictor of left ventricular remodeling and death. Survival, cardiac remodeling, and hemodynamic dysfunction are frequently proportional to the infarct size (88). The site of the occluding suture influences the size of the infarct and the outcomes of coronary occlusion, and it is difficult to identify the path of the LAD and the optimal ligation site in small-sized rats (49, 88).

### 2.2.2. Cryoinjury-induced MI

Cryoinjury is another technique used to create an MI rat model. It involves applying a cold probe to the surface of the heart, typically the left ventricle, to freeze and damage a small area of heart tissue. The resulting injury leads to a local inflammatory response and scar formation, mimicking the pathophysiological changes observed in human MI. This technique is commonly used to study the effects of different therapeutic interventions on cardiac repair and regeneration (89), as well as the underlying molecular and cellular mechanisms involved in cardiac remodeling after injury (90), evaluate the therapeutic effectiveness of biomaterials for cardiac repair in the MI model (91), and examine the effect of embryonic cardiomyocyte transplantation on HF progression (92).

According to Van Den Bos et al. (93), this is an ideal model for studying therapeutic interventions to restore heart function or cardiac regeneration following MI. They compared the results of myocardial injury created by cryoinjury with the CAL method and concluded that both resulted in a comparable loss of contractility and diastolic dysfunction, but the cryoinjury model demonstrated milder LV remodeling with no obvious heart failure. No obvious cardiac failure due to a minor necrotic disc-shaped lesion caused by the cryoprobe was observed. The generated lesion has cellular characteristics, such as coagulation necrosis of myocardium. Thus, it is an appropriate model for demonstrating myocardial repair (93, 94).

The pathophysiology of MI in the cryoinjury approach differs from that in other methods, such as LAD ligation, in that acute cell death occurs without accompanying ischemia. It is caused by mechanical stresses generated by the development of ice crystals in the intracellular and extracellular spaces, as well as in the vasculature (93). This technique has been employed in studies involving intracardiac cell transplantation for myocardial repair (89). Transplanted cells are easily injected at predetermined sites, and the presence of vascular reperfusion is favorable for cellular repair (93, 94).

#### 2.2.2.1. Surgical technique

Three consecutive exposures to a liquid nitrogen-cooled cryoprobe, a 6 mm stainless steel cylinder, resulted in acute LV MI. Blanching of the wall followed by hyperemia indicated the onset of MI in the heart. In addition, the cryoinjury region of an MI heart is distinguished by its pale appearance compared with the surrounding tissue (Figure 2) (89).

#### 2.2.2.2. Limitations

This method does not result in apparent HF following cryoinjury, which is most likely due to the smaller infarct size compared with coronary ligation. As a result, when an observable HF model is required, the cryo-infarction model is not a choice; in other words, cryo-infarction cannot replace the currently available HF models. Instead, it can be used as a model for evaluating medical treatments aimed at reducing cardiac remodeling and improving heart function after myocardial infarction, such as drugs that promote cardiac regeneration through progenitor cells or growth factors (93), and invasive surgical procedures involving thoracotomy, as in the LAD ligation technique (72).

### 2.2.3. Ischemia-reperfusion model

The IR rat model is a widely in used research to study the pathophysiology of ischemic injury and test potential therapeutic interventions. Its creation involves interrupting blood flow to a specific organ or tissue (ischemia) for a period of time and then restoring blood flow (reperfusion). In the case of the heart, a common approach is to temporarily occlude the coronary artery, induce myocardial ischemia, and then re-perfuse the tissue by removing the occlusion (95). This process results in a series of pathophysiological events, including oxidative stress, inflammation, and cell death, which can be studied to better understand the mechanisms of ischemic injury and identify potential therapeutic targets. The IR rat model is used to simulate ischemia-reperfusion injury that occurs in many clinical conditions, such as MI, stroke, and organ transplantation (96), and to evaluate the efficacy of human amniotic membrane mesenchymal stem cell-derived conditioned medium against IR injury (97).

Inducing MI in rodents with IR was originally tested in experimental *in vivo* organs before being used in dogs in 1988 (98). Initial apoptosis following hypoxia, as well as a smaller second wave of necrosis, causes an infarct after IR, which is, therefore, regarded as damage caused by reactive oxygen species and the opening of the mitochondrial permeability transition pores (95).

The implementation of early reperfusion in the clinical management of acute MI results in lower mortality and enhanced cardiac function (99). The period between occlusion and reperfusion ranged from 15 min to 2 h, with 30 min being the most common (100). However, there is insufficient information to support this conclusion. Some of the variances, as with the permanent CAL procedure, can be explained by factors such as operator experience and animal strain; however, the time of reperfusion adds another major level of variance and unpredictability to the outcome. As a result, 30 min after IR, the model may show infarct sizes of as low as 4%, indicating modest damage with no influence on heart function or eventual pathology, or as high as 30%, indicating minimal infarct size to significantly impair function (100, 101).

In all cases, the infarct size produced by IR was significantly smaller than that produced by the permanent CAL method because blood flow restoration rescues a portion of the affected area. One significant distinction between IR and permanent ligation (PL) is the secondary onset of reperfusion damage. This occurs as a direct result of the rapid return of blood flow to the damaged region, and is a secondary cause of cell damage and death after ischemia. In general, IR is more technically challenging than PL, resulting in smaller and more variable infarcts that frequently do not advance to other cardiovascular pathologies. However, it allows researchers to study the second wave of injury associated with blood reperfusion, which applies to clinical interventions in human acute MI patients but is not currently a therapeutic target (95).

#### 2.2.3.1. Surgical technique

The same applies to the MI model, with a difference in the ligation technique. After the heart was already visible, the LAD was temporarily ligated using a piece of tubing. The suture was cut, and the tubing was removed for reperfusion once the appropriate period of ischemia had passed (Figure 2) (39).

#### 2.2.3.2. Limitations

The most significant limitation of the IR model is that the majority of damage is still caused by ischemia, with reperfusion injury accounting for a considerably smaller second wave of post-MI injury. Therefore, reperfusion injury may have no apparent effect on the overall severity of MI (12). In addition, significant variations in the results and outcomes were mainly dependent on the IR time-course (102).

### 2.2.4. Aortic constriction model (pressure overload)

The AC model is a commonly used experimental model of left ventricular hypertrophy that involves partial constriction of the aorta to increase the pressure in the left ventricle. This model is used to study the mechanisms underlying cardiac hypertrophy and HF, to test potential therapeutic interventions (103), and to characterize the immunomodulatory response in a pressure overload model of HF (104).

Initially, banding had little or no effect on aortic flow, but as the animal grew, the relative severity of the constriction increased, resulting in heart hypertrophy, which has been utilized to mechanically replicate the cardiac consequences of aortic stenosis, systemic hypertension, and aortic coarctation in a variety of sites (3, 105).

The constriction may be thoracic, near the aortic origin (ascending AC [AAC]), or in the aortic arch between the first and second trunks (transverse AC [TAC]). The constriction can also be used in the abdominal aorta, either below or above the renal arteries, with the latter inducing hypertension due to renal hypoperfusion and concurrent LV hypertrophy. The main distinction among these models is the anatomic position of the constriction (106).

TAC and suprarenal AC cause a more gradual increase in pressure, hypertrophy, and HF, whereas AAC is frequently used to assess the effects of an early insult caused by pressure overload (107). The severity of the disease varies according to the species, age, and sex of the animal (108–110).

TAC surgery that reduces aortic diameter by 50%, causes a systolic pressure gradient of 50–60 mmHg between the aorta and the LV, resulting in clear echocardiographic evidence of LV hypertrophy and an increase in left atrial pressure around the eighth week (111). According to Weinberg et al. (109), after 18–20 weeks of compensatory LV hypertrophy, a subgroup of rats eventually showed reduced LV systolic pressure, higher LV volume, decreased ejection fraction, and clinical symptoms of overt congestive HF.

#### 2.2.4.1. Surgical technique

The anesthetized rats were placed in the supine position for TAC. Following the skin incision, the upper half of the sternum was separated in the midline using scissors, and the thymus was removed. The aortic arch was carefully dissected from surrounding tissues. A stylet (bent and blunted) 16G intravenous catheter was tied securely to the aorta between the brachiocephalic trunk and left common carotid artery using a 4.0 silk and then removed, creating partial AC. Sutures were used to close the sternotomy and skin incisions. Rats were extubated and placed in an incubator at 28–30°C for recovery (Figure 2) (45).

Rats were intraperitoneally injected with buprenorphine (0.1 mg/kg) for postoperative analgesia. In addition, the rats were administered oxytetracycline (500 mg/L of water) *via* drinking water for 7 days (112).

#### 2.2.4.2. Limitations

In the TAC model, although the onset of HF development differs significantly from that of patients with hypertension or aortic stenosis, the initiation of hypertension in this model is sudden and results in a 50% increase in LV mass within 2 weeks; thus, this is an ideal model to investigate intervention strategies that affect the development of cardiac hypertrophy (113).

AC (abdominal AC in the infrarenal and suprarenal positions) can also produce chronic LV pressure overload, which eventually leads to cardiac hypertrophy and dysfunction. The progression of this model to HF is more gradual, making it more appropriate for hypertension-related HF. Because it does not require chest opening or artificial breathing, it is more routinely utilized in rats than TAC (114, 115).

### 2.2.5. Two kidneys-one clip (2K1C) model (renal failure-induced hypertension, renovascular hypertension)

The 2K1C model is an experimental model of renovascular hypertension that involves placing a clip on one of the renal arteries to reduce blood flow to one of the kidneys. This reduction in blood flow stimulates the renin-angiotensin-aldosterone system, leading to increased blood pressure. This model was used to study the mechanisms of hypertension, test the efficacy of antihypertensive therapies (116), and study the significance of stem cell therapy in the remodeling of fibrotic kidney parenchyma (117).

The physiological function of the kidney includes maintaining electrolyte and fluid balance as well as secretion of renin, a key component of the renin-angiotensin system. Thus, its role in blood pressure regulation and the development of hypertension is widely acknowledged. Since Goldblatt et al. (118) created an elevation in blood pressure by partially closing the renal artery in dogs in 1934, many renal-generated hypertension models have been successfully established in rats, rabbits, sheep, and cats.

According to Weber et al. (119), within 2–4 weeks of clipping the kidney, the model is characterized by significant elevations in plasma renin activity, as well as elevated circulating angiotensin II concentrations and blood pressure. After 4 weeks, plasma renin activity and angiotensin II levels returned to near-normal levels, regardless of the presence of interstitial fibrosis in the heart, particularly around the intramural coronary arteries. Within a few months, a chronic phase developed, marked by increased plasma renin activity and myocardial perivascular and interstitial fibrosis (119).

Junhong et al. used 2K1C to simulate a rat model of diastolic dysfunction and studied its biochemical alterations using proteomic techniques. They found that diastolic dysfunction was observed in hypertensive rats 8 weeks after the operation, as evidenced by increased wall thickness, fibrosis, impaired relaxation, and increased chamber stiffness (54). Another experiment was conducted in our laboratory to induce renovascular hypertension in rats to study novel echocardiographic techniques and herbal medicines in this model (120).

#### 2.2.5.1. Surgical technique

**Figure 3:**
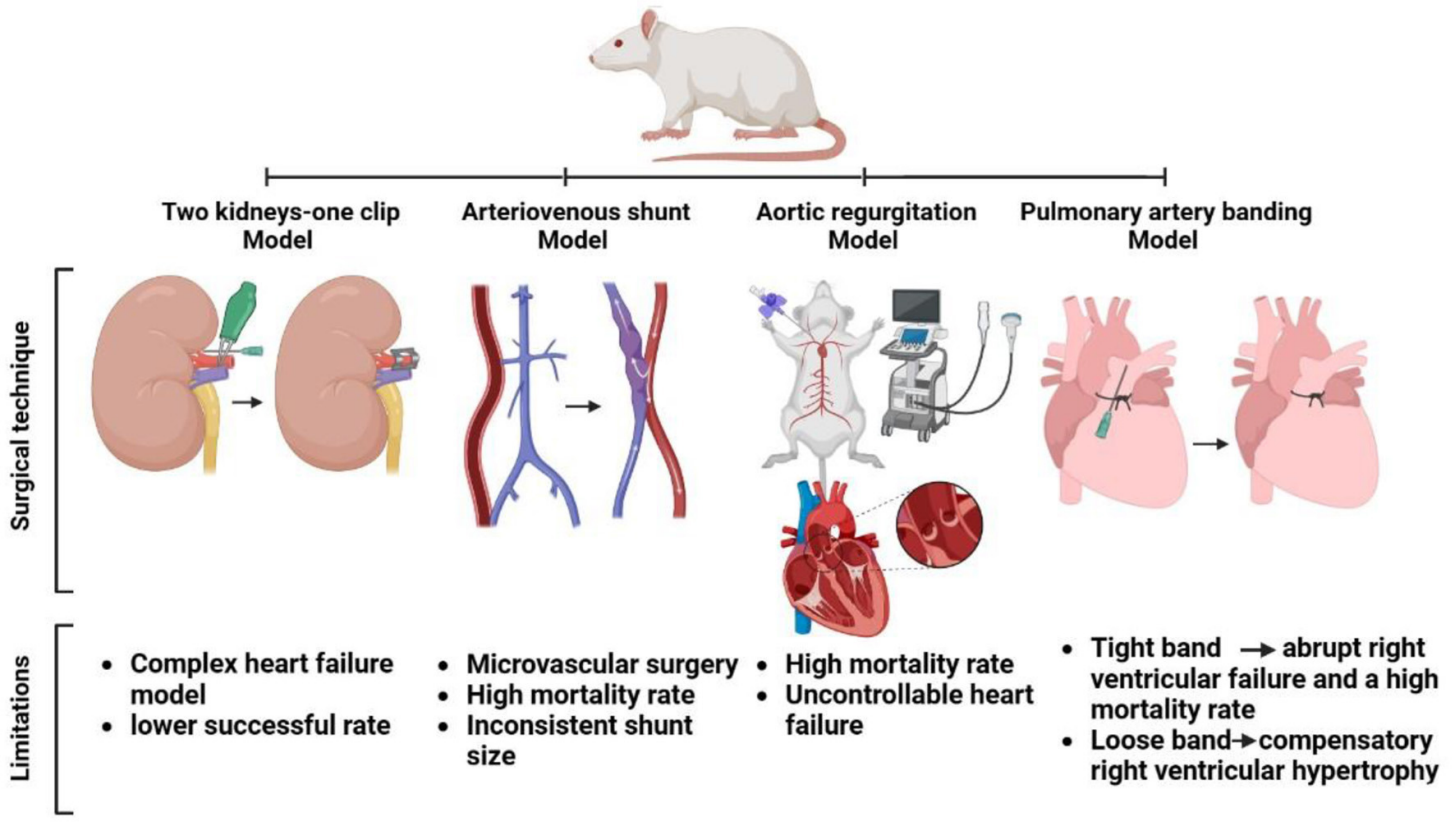
Surgical approach and shortcomings in some heart failure models in rats (two kidneys-one clip, arteriovenous shunt, aortic regurgitation, and pulmonary artery banding models).

The anesthetized rats are subjected to a flank abdominal incision to expose the left renal hilum, and the renal artery and vein are carefully identified by blunt dissection. To prevent vessel compression during clip placement, an insulin needle tip with an outer diameter of 0.23 mm is employed in each of the rats. A titanium vascular clip is also gently placed around the left renal artery. After that, the needle tip is carefully removed, the contents of the abdomen are gently returned to their original location, the abdominal wall and skin are sutured, and the animals are allowed to recover (Figure 3) (43, 55). Non-steroidal anti-inflammatory flunixin meglumine (2.5 mg/kg, subcutaneously) and antibiotic enrofloxacin (5 mg/kg, subcutaneously) can be administered as postoperative treatment (121).

#### 2.2.5.2. Limitations

Renovascular hypertension created by this model usually produces a complex HF model, in which myocardial hypertrophy is eccentric due to overloading and concentric due to hypertension that develops in addition to renal failure. Although this model somewhat mimics complex renal failure-induced HF to some extent, there are some issues regarding the success rate and rapid change in the geometry of the heart, which limit the study of detailed hemodynamic investigations of the heart. In a study by Ma et al. the feasibility of a novel IVPG assessment did not show significant benefits in this model within 8 weeks. However, his data revealed clear evidence of rapid changes in myocardial strain and the efficiency of a new medicine (salvianolic acid B) to ameliorate the pathological consequences of the heart in this model (120).

The 2K1C approach is not always successful in rats; for example, in the Dussaule experiment, 19 rats did not acquire hypertension, 27 had malignant hypertension, and 12 died; only 47 (45%) established stable hypertension (122). In addition, Amann et al. (123) found that after 14 months of uremia, ventricular hypertrophy in operated rats was not accompanied by an increase in the capillary number. Although this model undoubtedly enables the analysis of hypertension following renal failure, it is difficult to determine how it may be used for human essential hypertension (124).

### 2.2.6. Arteriovenous shunt model (volume overload)

The AVS model is an experimental model that involves the surgical creation of a direct connection between an artery and vein, bypassing the capillary bed. This results in increased blood flow and pressure in the vein, mimicking the hemodynamic changes observed in certain pathological conditions, such as arteriovenous fistulas. The AVS model was used to study the effects of increased blood flow and pressure on vascular function and to test potential therapeutic interventions (32).

Aortocaval fistula (ACF)-induced chronic volume overload in rats is a well-studied rodent HF model (106). This model is simple and reliable, and it features several crucial aspects of human HF, including a gradual change from the asymptomatic to the decompensated phase, considerable neurohumoral activation (125), fluid retention, and changes in myocardial phenotype typical of HF (126).

An artificial shunt between the abdominal aorta and the inferior vena cava causes a significant increase in cardiac output and venous return, which causes compensatory, initially asymptomatic ventricular hypertrophy (127), prolonged hemodynamic overload, redistribution of cardiac output, and activation of the neurohumoral response, causing HF to appear 8–10 weeks after ACF induction (57).

AVS have been used to cause volume overload, dilated cardiomyopathy, and HF in rodents (128). Despite the limitation of requiring laparotomy, the more recent aortocaval shunt technique is a comparably faster and easier way to induce HF with good survival rates (3, 129).

The hemodynamic data in these models suggest a persistent increase in the LV diastolic volume. The Frank–Starling mechanism is responsible for maintaining a high output status in the early stages following the development of the AV shunt. This variable represents an abrupt increase in wall stress caused by volume overload, whereas changes in LV end-diastolic pressure indicate that the development of cardiac hypertrophy and dilation of the cardiac chamber tend to regulate wall stress (6).

#### 2.2.6.1. Surgical technique

Flaim et al. established a HF rat model of a chronic AVS between the abdominal aorta and the inferior vena cava, and surgical introduction of an arteriovenous fistula between the abdominal aorta and the inferior vena cava at a point ~5 mm caudal to the left renal vein was used to induce a high cardiac output state. After general anesthesia, a midline incision in the abdominal wall was made to expose the peritoneal cavity, and the abdominal aorta and vena cava were exposed and isolated for ~20 mm before all branches were occluded using bulldog clamps A 10 mm segment of the aorta and vena cava were isolated under a dissecting microscope by placing two bulldog clamps across the main vessels, and openings of approximately comparable size (width, 1 mm) were made through the medial walls at the midpoint of the isolated segments. Three interrupted microsurgical sutures (9-O Ethilon) were used to unite the opposing sides of the two apertures; the clamps were removed, and the patency of the fistula was visually confirmed by the presence of mixed arterial blood in the vena cava. After closing the abdominal incision, the animal was allowed to recover (Figure 3) (125, 130).

On the other hand, Garcia and Diebold developed a simple, rapid, and effective method for exposing the vena cava and abdominal aorta by opening the abdominal cavity with a midline incision, placing a 18-gauge needle into the abdominal aorta and progressing through the medial wall into the vena cava, constricting the aorta is momentarily below the origin of the right renal artery, and quickly repairing the aortic puncture using a cyanoacrylate glue. To confirm the patency of the shunt, a pulsatile flow of oxygenated blood into the inferior vena cava is visually observed. A standard method is used to close the abdominal cavity using an absorbable suture (131).

#### 2.2.6.2. Limitations

In rats, an AV fistula is formed by a side-to-side anastomosis of the aorta and vena cava (125) or by end-to-side anastomosis of the left iliolumbar vein (56). Both operations necessitate microvascular surgery, and the circulatory system is occluded for 15–30 min. Furthermore, because these surgical procedures take 40 mins to complete, mortality rates range from 47 to 76% (56, 125). In addition, shunt size and hypertrophic and hemodynamic characteristics have been inconsistent (13).

### 2.2.7. Aortic regurgitation model (volume overload)

AR models are often created through surgical interventions such as aortic valve leaflet perforation or cusp removal. These procedures can lead to increased retrograde blood flow, resulting in AR. The severity of regurgitation can be assessed using various imaging techniques, such as echocardiography or MRI. These models can be used to study the pathophysiology and potential treatments of AR (132) and to study biological and tissue-engineered valvular and cardiovascular grafts *in vivo* (133).

AR is another volume overload model of HF that is induced by ventricular volume regurgitation and is thus directly related to the severity of aortic insufficiency. Mild AR causes only minor volume overload, whereas severe AR causes considerable LV volume overload and increasing chamber dilatation. AR can be classified as compensated or decompensated. In compensated AR, the LV first responds to volume overload by eccentric hypertrophy, preserving LV diastolic compliance and allowing LV filling pressures to stay normal or mildly elevated despite a substantial regurgitation volume. Decompensated AR is defined as LV systolic dysfunction and poor LV diastolic compliance as a result of hypertrophy and fibrosis, resulting in excessive filling pressures and HF (6, 134).

Although not the most frequently encountered valvular disease, it has been estimated based on the findings of the Framingham study that 13% of the population suffers from some degree of AR (135). While mild AR normally does not cause any significant problems, the disorder can grow silently for decades and worsen. This stealthy progression results in increased LV dilatation, hypertrophy, and, finally, HF (136, 137).

#### 2.2.7.1. Surgical technique

AR is established in anesthetized animals by exposing the right carotid artery through a right lateral neck incision. The distal common carotid artery is ligatured using a 4.0 nylon suture, followed by a arteriotomy to allow the insertion of a 0.9-mm guide wire. The thorax is scanned with an echocardiographic probe to obtain a clear view of the left ventricle, aortic valve, and ascending aorta, which is equal to a parasternal long-axis view in standard human echocardiography. Under continuous echocardiographic observation, an arterial leader catheter is moved retrogradely toward the aortic valve, and the position and passage of the catheter through the aortic valve leaflet and into the left ventricle are guided by the sonographer; an acute AR is caused by a tear in the leaflet (Figure 3) (138).

The following echocardiographic criteria were used to determine AR at the time of surgery: the color Doppler ratio of regurgitation jet width to left ventricular outflow tract obstruction diameter was 50–70%, and pulsed-wave Doppler proved reversed diastolic flow in the abdominal aorta (139). When the echocardiographic criteria determined that the severity of the regurgitation jet in the abdominal aorta was insufficient, leaflet perfusion was repeated. After the AR was established, the proximal carotid artery was ligated using 4.0 nylon sutures (140). In the first few hours following surgery, the animals were observed for any signs of respiratory distress that could indicate severe HF. They were weighed daily to check for excessive weight gain, which could be a sign of pending HF (138).

#### 2.2.7.2. Limitations

This rat model has various drawbacks in terms of AR. One of the most serious complications is the extremely high mortality rate associated with acute AR secondary to HF. Multiple aortic valve leaflet perforations can cause serious valve damage, uncontrollable HF, and death. Therefore, wire perforations should be performed while echocardiography is being monitored, and repeated perforations should be avoided (140).

Another challenge is the wire size of the perforations. Some researchers prefer thicker wires (0.9 mm), which can result in multiple leaflet injuries with severe AR, while others prefer thinner wires (0.3 mm), which result in relatively moderate AR (138, 141). Thus, a wire diameter of 0.6 mm may be more acceptable for creating a modest AR model (6).

### 2.2.8. Pulmonary artery banding model (right ventricle pressure overload models)

PAB is a surgical model used in rats to induce right ventricular (RV) pressure overload and study the development of RV hypertrophy and HF. In this model, a band is placed around the pulmonary artery, restricting blood flow to the lungs and causing an increased RV afterload. The severity of RV pressure overload can be adjusted by varying the tightness of the band. PAB is commonly used in cardiovascular research to investigate the molecular and cellular mechanisms involved in RV hypertrophy and HF to test potential therapies for these conditions (28) and to explore the efficacy of stem cell therapy for RV failure in pulmonary arterial hypertension (142).

Pulmonary arterial hypertension (PAH) is a chronic and frequently fatal condition (143). Although the primary pathology originates in the pulmonary vasculature, mortality is determined by RV remodeling, dysfunction, and eventual failure (144). Several animal models of RV pressure overload and PAH have been developed to study the pathophysiology of PAH and RVs, as well as their response to prospective treatments. PAB, Sugen-5416 plus hypoxia (SuHx)-induced PAH, and monocrotaline (MCT)-induced PAH are some of the models used (145–148). Several studies have demonstrated that these models produce distinct RV responses in terms of adaptive RV hypertrophy in the PAB model, in contrast to the maladaptive failure in the SuHx and MCT models (28, 149). Several characteristics of maladaptive RV remodeling in the PAH model have been proposed, including RV dilation, reduced function, fibrosis, and capillary rarefication (29, 150). However, as of the confounding effects of potentially altered pulmonary vascular resistance, hypoxia, molecular modulation (e.g., VEGF inhibition), or toxins on RV function, the MCT and SuHx models cannot be used to investigate isolated RV effects of potential therapies, and the PAB model is relevant in this regard (29, 151, 152).

PAB involves constricting the pulmonary artery using a band or clip to increase the workload on the right ventricle and simulate the effects of pulmonary hypertension. A pre-adjusted hemostatic clip is the most widely used approach in small animal models of rats and mice (48, 153, 154), or a ligature tightened around the pulmonary artery (155–157). Both procedures are effective; however, the clipping approach may be easier to learn and more reproducible, whereas the ligature method does not use metal, making it better suited for MRI or ultrasound evaluation of pulmonary artery flow (158). The banding method has the advantage of allowing for accurate titration of afterload to produce RV hypertrophy, compensated RV failure, or decompensated RV failure owing to the precise diameter of the band/clip (159, 160), as evidenced by hypertrophy with preserved hemodynamics, altered hemodynamics without extracardiac symptoms of RV failure, and altered hemodynamics with extracardiac signs of RV failure (158).

#### 2.2.8.1. Surgical technique

The anesthetized animals are mechanically ventilated after intubation using a volume-controlled respirator and oxygen-enriched room air. After induction left thoracotomy, the pulmonary artery (PA) is gently torn free from the aorta using a silk thread that is threaded beneath the PA, then an 18-gauge needle is threaded alongside the PA, and the suture is securely tied around the needle and swiftly released, leaving a fixed, constricted aperture in the lumen equal to the needle's diameter. The combination of a fixed banding around the PA and the animal's growth results in dramatically elevation RV afterload over time (Figure 3) (28, 161, 162). In another technique using a clip applier with a stopper, a small clip is half-closed around the PA, and blood flow *via* the PA is restricted to the inner segment of the half-closed clip (59). Buprenorphine (15 g/kg sc) is used to relieve postoperative pain (28).

#### 2.2.8.2. Limitations

A challenge with the banded model is to include RV failure, rather than simply a well-adapted hypertrophic RV. The difficulty is that a tight band causes abrupt RV failure and mortality in adult animals, whereas a loose band causes compensatory RV hypertrophy. To overcome this, most models begin operations with weaning. This causes stenosis to worsen as the animal develops, allowing for catastrophic RV failure over time (158, 163).

## Author contributions

Review design: AF, AM, and RT. Investigation: AF, AM, HH, and AElh. Data collection: AF, AElf, and HH. Writing and drafting: AF, AM, AElh, and AElf. Critical editing: AM. Supervision: RT. All authors reviewed and edited the final version.
